# Characterization of Hybrid Oil Palm Empty Fruit Bunch/Woven Kenaf Fabric-Reinforced Epoxy Composites

**DOI:** 10.3390/polym12092052

**Published:** 2020-09-09

**Authors:** Farah Hanan, Mohammad Jawaid, Md Tahir Paridah, Jesuarockiam Naveen

**Affiliations:** 1 Laboratory of Bio Composites Technology, Institute of Tropical Forestry & Forest Product (INTROP), Universiti Putra Malaysia, UPM Serdang, Selangor 43400, Malaysia; faraluf@gmail.com (F.H.); parida.introp@gmail.com (M.T.P.); 2 School of Mechanical Engineering, Vellore Institute of Technology, Vellore 632014, India; naveen.j@vit.ac.in

**Keywords:** hybrid composites, oil palm fiber, kenaf fiber epoxy, mechanical properties, physical properties, morphological properties

## Abstract

In this research, the physical, mechanical and morphological properties of oil palm empty fruit bunch (EFB) mat/woven kenaf fabric-reinforced epoxy composites have been investigated. The oil palm EFB/woven kenaf fabrics were varied, with weight ratios of 50/0 (T1), 35/15 (T2), 25/25 (T3), 15/35 (T4) and 0/50 (T5). The composites were fabricated using a simple hand lay-up technique followed by hot pressing. The result obtained shows that an increase in kenaf fiber content exhibited higher tensile and flexural properties. On the other hand, the opposite trend was observed in the impact strength of hybrid composites, where an increase in kenaf fiber content reduced the impact strength. This can be corroborated with the physical properties analysis, where a higher void content, water absorption and thickness swelling were observed for pure oil palm EFB (T1) composites compared to other samples. The scanning electron microscopy analysis results clearly show the different failure modes of the tensile fractured samples. Statistical analysis was performed using one-way ANOVA and shows significant differences between the obtained results.

## 1. Introduction

In recent years, the incorporation of natural fibers in polymer composites has received much attention from researchers and various industries due to their superior mechanical performance. This trend has led to the use of natural fibers for reinforcement rather than relying on synthetic fibers [1]. Natural fibers, including kenaf, coir, oil palm, banana, kapok flax, hemp, jute, and sisal, exhibit superior mechanical properties compared to synthetic fibers. Aside from the various advantages of natural fibers such as their non-abrasive, low cost, low density, reduced energy consumption and biodegradability properties, they exhibit a few impediments such as moisture absorption and thermal degradation [2,3,4]. However, different surface modifications can overcome these drawbacks [5,6].

In Malaysia, the overproduction of agricultural commodities such as oil palm fiber, their byproducts (empty fruit bunch (EFB), oil palm trunks and oil palm fronds) are produced in quantities in the order of millions of tons per annum [7]. Malaysia, which is a tropical country, has hot climate and weather throughout the year, which encourages the cultivation of oil palm. Around 4.85 million hectares of oil palm industry contributes about RM 60 billion in terms of export revenues in 2010 and has grown rapidly in recent years. Malaysia has produced about 40,000–50,000 tons of crude palm oil per day and, indirectly, it has generated a vast quantity of palm biomass, which consists of two different sources, namely plantations (trunks and fond) and mills (EFB, palm kernel shell, palm oil mill effluent). The palm oil sector appears to be one of the potential energy sources due to its abundance and Malaysia is working towards the realization of the palm oil sector for producing value-added products and biochemical’s to increase the business opportunities for the palm oil industry. Empty fruit bunch, or EFB, has been widely used in the production of various products such as furniture and mattresses, in erosion control, paper production, sofa/car seats and also landscaping [8].

The kenaf fiber, which is comparatively very cheap and commercially available in Southeast Asian countries, has been utilized as a reinforcing material in polymeric composites. Kenaf (*Hibiscus Cannabinus*) is a single, straight and branchless stalk made up of core and bastfibers of 75%–60% and 25%–40%, respectively. Bast fiber is extracted from the outer layer of the kenaf plant, whereas the inner portion is used for obtaining core fiber through a retting process. Kenaf consists of cellulose (45%–57%), hemicelluloses (21.5%), lignin (8%–13%) and pectin (3%–5%) [9]. Kenaf fiber exhibits good mechanical properties, a low density, which is nonabrasive during processing, a good specific strength and compatibility with polymers such as epoxy and phenolic resin. Kenaf fiber composites can be efficiently utilized for many light-duty applications, such as in the automotive, textile, food packaging, sports and furniture industries [10].

Hybridizing high modulus and low modulus fiber takes advantage of their superior properties in terms of both reinforcement and results. Mirbagheri et al. [11] found that kenaf fiber/wood flour/polypropylene hybrid composites showed improved mechanical properties after adding the maximum proportion of kenaf fibers. Atiqah et al. [12] developed hybrid composites with glass and kenaf fabric using a sheet-molding compound process. The hybrid composites exhibited better mechanical properties compared to kenaf fiber-based composites. The mechanical and water uptake behavior of hybridized kenaf/pineapple leaf fiber-reinforced high-density polyethylene composites were studied by Aji et al. [13] and they observed that kenaf/pineapple fiber composites showed a better impact strength and a reduction in water uptake compared to pineapple fiber composites. Jawaid et al. [14] reported that hybrid composites consisting of oil palm/jute exhibited excellent mechanical properties compared to monolithic composites. Ewulonu and Igwe reported that increases in EFB filler content in high density polyethylene composites improved the hardness and specific gravity [15]. Rozman et al. [16] investigated the mechanical properties of oil palm/glass fiber hybrid composites and found that increasing the oil palm fiber content (10%, 30% and 40%) in the matrix reduced the tensile strength and enhanced the modulus. Yusoff et al. [17] observed that the addition of 5% Vf (volume fraction of fiber) in EFB in epoxy composites can increase the tensile modulus. However, a further increase in EFB content reduced the tensile modulus of the composites. The flexural modulus of oil palm fiber/polypropylene composites attempted by Khalil et al. [18] presented that oil palm fiber can increase the flexural modulus of pure polypropylene resin but reduces the flexural strength.

The overall performance of natural fiber-reinforced polymeric composites depends on the natural chemicals presents in the fiber, as well as physical and mechanical properties of natural fiber and polymer matrix materials [19]. Mostly, the structural load is carried by the fiber, whereas the shape, surface and environmental resistance is carried by the matrix [20]. The novelty of the present research work is the efficient utilization of EFB agro waste to produce a sustainable composite panel for non-load bearing applications. Moreover, the present research investigates the mechanical, morphological and physical properties of randomly oriented oil palm EFB mat/woven kenaf fabric epoxy composites.

## 2. Materials and Methods

### 2.1. Materials

Oil palm EFB fabric was supplied by HK Kitaran Sdn. Bhd. (P.Pinang, Malaysia). Woven kenaf fabric was procured from ZKK Sdn. Bhd. (Selangor, Malaysia) and the properties are presented in Table 1. In this study, resin (epoxy resin types DER-331), curing agent (epoxy hardener joint amine 905-3s) and silicon spray were supplied by TAZDIQ Engineering Sdn. Bhd (Selangor, Malaysia).

### 2.2. Fabrication of Hybrid Composites

For the preparation of bi-layer hybrid composites, a stainless steel mold with dimensions of 300 mm × 300 mm × 5 mm was used with the hand-lay-up technique for making the test sample. A releasing agent (silicon spray) was coated with a thin layer on the mold. Epoxy resin and hardener (2:1) were mixed for 15 min. Bi-layered hybrid composite samples were made with different weight ratios of oil palm EFB and kenaf such as 4:1, 1:1, and 1:4 with a total fiber loading of 50 wt%. The oil palm EFB and kenaf mats were stacked alternatively, and then the resin mixture was poured over each layer inside the mold. Entrapped air bubbles were removed using a hand roller. The mold was closed for the curing process using a hot press machine for 15 min at 120 °C. Eventually, to avoid warping the bi-layer, hybrid composites were cooled in a cold press for 5 min under a constant pressure of 250 bars. Pure oil palm EFB and kenaf fiber composites were also prepared as a control sample. Table 2 shows the formulations of oil palm EFB/woven kenaf fiber-reinforced epoxy-based bi-layer hybrid composites.

## 3. Characterizations

### 3.1. Tensile Test

Tensile testing was carried out using a universal testing machine, (INSTRON 4201, Instron, Norwood, CO, USA) with a 100 kN capacity load cell in accordance with ASTM D 3039 standard. Five samples (120 mm × 20 mm × 5 mm) were tested with a loading rate of 5 mm/min.

### 3.2. Scanning Electron Microscopy (SEM)

The morphology of tensile fractured specimens of oil palm EFB, kenaf and bi-layer hybrid composites was analyzed by scanning electron microscope (Hitachi, Krefeld, Germany). The tensile fractured specimens were sputter-coated with thin layer of gold to improve the visual inspection.

### 3.3. Flexural Test

A flexural test was carried out by using a universal testing machine, (INSTRON 4201, Instron, Norwood, CO, USA) with a 100 kN capacity load cell according to ASTM D 790 standard. The dimensions for test samples were120 mm × 20 mm × 5 mm with a crosshead speed of 5 mm/min. 

### 3.4. Impact Test

An impact test was performed according to ASTM D 256 standard using the Gotech GT-7045-MD model (Gotech, Taichung city, Taiwan). The Izod method was carried out using notched samples with dimensions of 70 mm × 15 mm × 5 mm. Five identical samples were tested and the results were tabulated.

### 3.5. Void Content

Voids in hybrid composites were determined as per ASTM-D-2734-70. The void content was calculated by using Equations (1)–(4).

*r = MF/MB × 100* (1)

*R = 100 – r* (2)

*Td = 100/(R/D + r/d)* (3)

*Void Content = 100(Td − Md)/Td* (4)

where *R* is the weight % of the resin in the composite, *r* is the weight % of the reinforcement in the composite; *MF* is the mass of the fiber, *MB* is the mass of the composite, *D* is the density of the resin matrix, *d* is the density of the reinforcement, *Td* is the theoretical density and *Md* is the measured density.

### 3.6. Density

Density was measured by using the ASTM D1895 standard. The density of the samples was calculated by using Equation (5):

*Density(g/cm^3^) = m/v* (5)

where *m* is the mass of the composites, and *v* is the volume of the composites.

### 3.7. Dimension Stability Test

Water absorption tests were carried out according to ASTM D-570 specifications. Flexural specimens were cut from the compression-molded plates and used for the measurements of water absorption and thickness swelling. After vacuum drying at 80°C for 24 h to a constant weight and a precision of 0.001 g, the weight of specimens before water immersion (*W_d_*) was measured, while balance and thickness were measured with a thickness gauge (*T*_0_). The specimens were immersed in water at room temperature. Five samples of each type of composite were immersed in distilled water at room temperature. The percentage of water absorption was calculated from Equation (6) and the thickness swelling from Equation (7):

(6) $$Mt\left(\%\right)=\frac{w_{w}-w_{d}}{w_{d}}\;\times 100$$

where *W_d_* and *W_w_* denote the weight of the dry material (the initial weight of materials before water immersion) and the weight of materials after water immersion, respectively. The specimens were immersed until they were saturated.

(7) $$Mt\left(\%\right)=\frac{T_{w}-T_{0}}{T_{0}}\;\times 100$$

where *T*_0_ and *T_w_* are the thicknesses (mm) of the sample before and after immersion, respectively.

## 4. Results and Discussion

### 4.1. Tensile Properties

**Figure 1:**
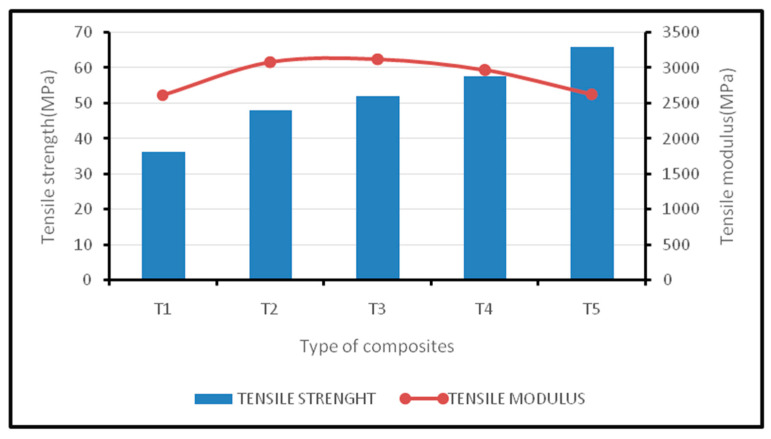
Tensile strength and modulus of oil palm EFB/woven kenaf fiber-reinforced epoxy-based bi-layer composites.

The tensile properties of fiber-reinforced composite materials mainly depend upon the following factors: tensile strength of the fiber; interfacial adhesion of fiber/matrix; aspect ratio of fiber; orientation of the fibers; their dispersion into the matrix [23]. The fiber/matrix interfacial adhesion plays a significant role in the mechanical properties of composites. This research has focused on hybridizing the stronger and stiffer kenaf fiber with the oil palm EFB fiber. It has been shown in Figure 1 that kenaf composites (T5) exhibit the highest values with 65.9 MPa compared to EFB composites (T1). This is mainly due to the longitudinally oriented stronger and stiffer kenaf fibers, which improve the load carrying capacity and mechanical strength. Alavudeen et al. [24] also found that tensile strength is higher when a fiber of greater strength is oriented longitudinally so as to bear the tensile load along the direction of the fiber. 

In hybrid compositions, the tensile strength of oil palm EFB/kenaf woven fiber-reinforced epoxy based bi-layer composites has been improved by increasing the kenaf ratio in the hybrid composite. As expected, T4hybrid composites exhibit a higher strength with a value of 55.7 MPa among the hybrid composites; these values are close to the T5 composite but, in the tensile modulus, are shown to be slightly lower than T3 hybrid composites with a value of 2972.8 MPa. The tensile strength and tensile modulus of T2 hybrid composites are higher by 132.7% and 117.8%, respectively, while the T3 hybrid composites achieve 143.4% higher values for tensile strength and 119.6% higher values for tensile modulus than the corresponding values of T1 composites. 

In the case of bi-layered composites, the load from the kenaf fiber mat is not directly transferred to the oil palm fibers, which leads to gradual failure during the loading process. Since the failed kenaf fiber mats are still able to carry the load, this prolongs the failure of the EFB fiber. Asim et al. [21] developed the fiber bundle theory, which uses a weaker fiber break first at normal strain, while a stronger fiber holds the matrix in weaker fibers. Although fibers have different characteristics, they still distribute the load and contribute to making a hybrid composite stiffer. In this case, EFB fibers are referred to as the weaker fiber, while kenaf fibers are referred to as the stronger fiber based on their tensile properties. Jawaid et al. [14] also found a similar result while hybridizing jute and kenaf (4:1) in epoxy matrix. The incorporation of EFB in the epoxy matrix showed comparatively lower tensile properties. This may be due to the weak interface, which results in the non-uniform loading of micro fibrils [25]. Furthermore, it reduced the load carrying capability of the macro EFP fiber Hence, the incorporation of EFB as a major reinforcement becomes ineffective compared to synthetic fibers and other natural fibers.

Table 3 presents the ANOVA test results for the tensile modulus. The variance in the tensile modulus was organized into two groups: within the group (WG) and between the groups (BG). F-ratio is the ratio between the mean square (BG) to the mean square (WG). A statistically significant difference was found between the mean tensile modulus, since the *p*-value is less than 0.05 with a 95% confidence level. Table 4 presents the ANOVA test results for tensile strength. The variance in tensile strength was organized into two groups: within the group (WG) and between the groups (BG). F-ratio is the ratio between the mean square (BG) to the mean square (WG). A statistically significant difference was found between the mean tensile strength, since the *p*-value is less than 0.05 with a 95% confidence level.

### 4.2. Scanning Electron Microscopy (SEM)

**Figure 2:**
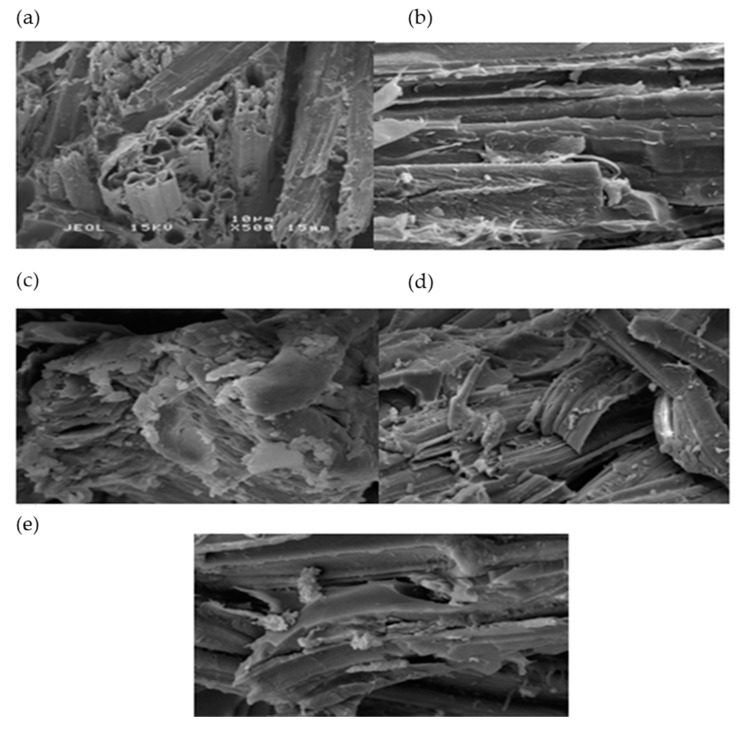
Scanning electron micrographs of tensile fracture of T1 composite (**a**), T5 composite (**b**), T2 hybrid composite (**c**), T3hybrid composite (**d**), T4 hybrid composite (**e**), scale bar = 10 μm.

The SEM micrographs of tensile fractured T1 composites (a), T5 composites (b), T2 hybrid composites (c), T3 hybrid composites (d), T4hybrid composites (e) are shown in Figure 2. The SEM micrograph for the EFB composites (a)clearly shows weak interfacial interactions between the EFB fibers and the polymeric matrix compared to the kenaf woven fiber (b) composite, which reveals an even distribution of the fiber and a good standing interfacial bonding, which contributes to an effective stress transfer from the polymer to enable reinforcement. Evidence from the SEM micrograph also reveals significant fiber pullout and micro cracks in the polymeric matrix, which leads to weak interfacial interactions between the EFB fiber and epoxy matrix. 

Hybrid composites exhibiting a homogeneous distribution of fiber, fiber pullout and void content can be easily seen on the fracture surface [21]. Figure 2c,d which are T2 and T3 hybrid composites respectively showed the non-uniform fiber dispersion. Debonding of EFB fiber at the interface is also clear evident from Figure 2c,d. Phenomenon of fiber pullout also can be observed from Figure 2c,d. From Figure 2e it is understood that kenaf fiber exhibited very less fiber pullout because of higher modulus of kenaf fiber. Moreover, it has been observed that kenaf fiber showed a more densified and compact structure compared to oil palm fiber. Alavudeen et al. [24] also observed that kenaf fiber composites exhibited better mechanical properties due to their dense fabric architecture. Shibata et al. [26] found that the orientation and structure of the fiber/fabric influences the properties of the composites, while hybridizing kenaf and bagasse in a biodegradable resin Therefore, the interfacial structure of T4 hybrid composites showed good interfacial bonding, and thus additional kenaf fiber improved the interfacial strength compared to other hybrid composites that had a lower tensile strength caused by heterogeneous fiber distribution.

### 4.3. Flexural Properties

**Figure 3:**
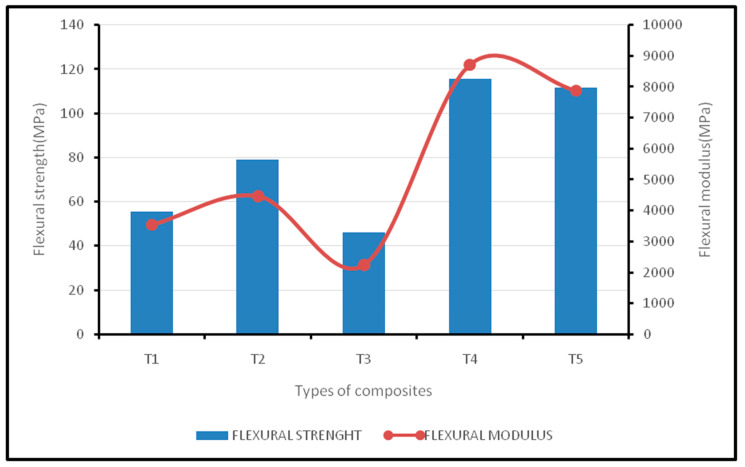
Flexural strength and modulus of oil palm EFB/kenaf woven fiber-reinforced epoxy-based bi-layer composites.

The flexural properties of oil palm EFB/ woven kenaf fiber based bi-layer hybrid epoxy composites are shown in Figure 3. The results show that the flexural strength and modulus of T4 hybrid composites are slightly higher than T5 composites, with values of 115.8 MPa and 8724.7 MPa, respectively. T5 composites were recorded with values of 111.68 MPa for flexural strength and 7900.8 MPa for flexural modulus. These two types of composites exhibited good compatibility with epoxy resin in terms of flexural properties. A common phenomenon was shown where the flexural strength and modulus declined while the EFB fiber content increased. The T2 hybrid composite had values of 79.05 MPa and 4484.7 MPa, respectively, for flexural strength and modulus, while T3 hybrid composites had values of 46.05 MPa for flexural strength and 2248.7 MPa for flexural modulus.

Rozman et al. [16] mentioned that, for randomly oriented irregular fibers such as EFB fiber, their capability to bear the stress is generally poor and the effect will be amplified if the EFB fiber proportion is increased. Overall, a similar trend has been shown for flexural strength and modulus. Zuhri et al. [17] declared that EFB fiber decreased the flexural modulus of epoxy polymer. Jawaid et al. [14] reported their experience of using EFB fiber in a hybrid composite reinforcing jute fiber, indicating a reduction in flexural properties with an increasing EFB fiber content. The lowest values were found for the T1 composite—45.1 MPa for flexural strength and 3557.9 MPa for flexural modulus, respectively.

Table 5 shows the ANOVA test results for the flexural modulus. The variance in the tensile modulus was organized into two groups: within the group (WG) and between the groups (BG). F-ratio is the ratio between the mean square (BG) to the mean square (WG). A statistically significant difference was found between the mean flexural modulus, since the *p*-value is less than 0.05 with a 95% confidence level. Table 6 presents the ANOVA test results of flexural strength. The variance in flexural strength was organized into two groups: within the group (WG) and between the groups (BG). F-ratio is the ratio between the mean square (BG) to the mean square (WG). A statistically significant difference was found between the mean flexural strength, since the *p*-value is less than 0.05 with a 95% confidence level.

### 4.4. Impact Strength

**Figure 4:**
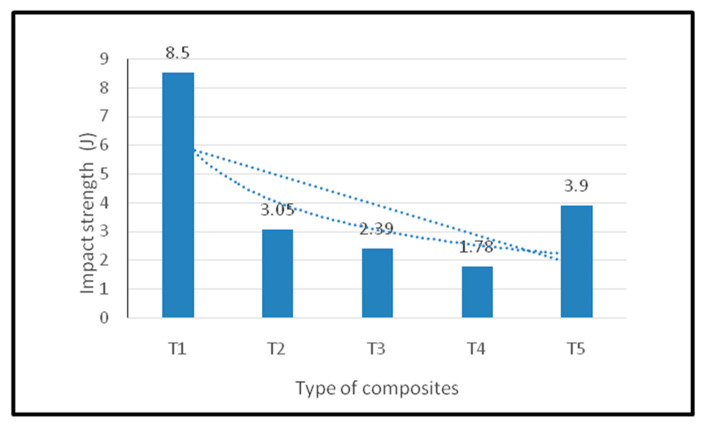
Impact strength of oil palm EFB/woven kenaf fiber-reinforced epoxy-based bi-layer composites.

The impact strength of kenaf/oil palm EFB hybrid composites is shown in Figure 4. It was observed that theT1 composite exhibited the highest impact strength of 8.05 J compared to other composites. T5 composites indicate the second highest, 3.9 J, followed by T2 composites (3.05 J), T3 (2.39) and T4 (1.78). Both T1 and T5 are single composites and they had a better impact of strength than hybrid composites. Similar results were observed by other researchers while using oil palm EFB and jute fiber-based hybrid composites. This is mainly attributed to the fiber orientation and chemical composition of the oil palm EFB [27,28]. Moreover, it can be explained with another phenomenon where randomly oriented EFB forms a moderate interfacial interaction with epoxy, which is essential to achieving a higher impact strength.

Table 7 shows the ANOVA test results for impact strength. The variance in the tensile modulus was organized into two groups: within the group (WG) and between the groups (BG). F-ratio is the ratio between the mean square (BG) to the mean square (WG). A statistically significant difference was found between the mean impact strength, since the *p*-value is less than 0.05 with a 95% confidence level. 

### 4.5. Void Content and Density

Table 8 shows the void content and density of oil palm EFB/woven kenaf fabric-reinforced epoxy composites. Oil palm EFB (T1) indicates the highest void content of 8.40% and the lowest density at 1.02 g/cm^3^, respectively. Fiber–matrix dispersion and the wetting of the fibers occurs clearly in oil palm EFB composites. Jawaid et al. mentioned that, during the process of matrix impregnation, all the air entrained within the fiber was incompatible with the matrix, which is the most common cause of voids. Voids reduce the variation in mechanical properties, including interlaminar shear strength, longitudinal and transverse flexural strength and modulus, longitudinal and transverse tensile strength and modulus, compressive strength and modulus and fatigue resistance. The addition of kenaf fiber into oil palm EFB fiber reduced the percentage of void content, attributed to the higher density of kenaf fiber compared to oil palm fiber. Epoxy resin is more compatible with kenaf due to the tightly packed matt fiber, which allows a slightly smaller amount of voids and a lower density percentage in the hybrid composites. Compared to oil palm fiber, the mat is loosely packed and porous, leading to the squeezing out of large amounts of resin during the pressing and molding process.

### 4.6. Water Absorption

**Figure 5:**
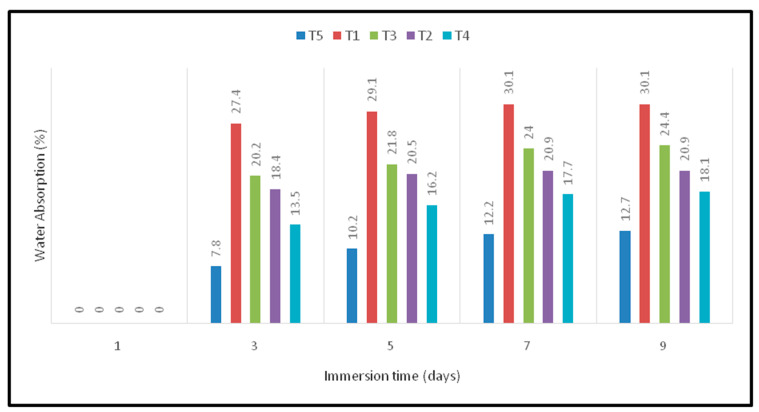
Water absorption (%) of oil palm EFB/kenaf woven fiber-reinforced epoxy-based bi-layer composites.

Among the factors related to moisture diffusion are the volume fraction of the fiber, the viscosity of the matrix, voids, humidity and temperature [29]. In natural fibers, the main chemical entity to attract water molecules is the hydroxyl group, which absorbs water during the formation of hydrogen bonding [26]. Natural fibers are prone to moisture and water absorption because of the pressure of constituent materials such as cellulose and hemicelluloses, which are hydrophilic [30]. Figure 5 presents the water absorption of oil palm EFB/woven kenaf fabric-reinforced epoxy composites. This clearly shows that water absorption increased with immersion time. The high porosity on the surface of oil palm EFB composites exhibits the highest water absorption in T1 composites. Hassan et al. [31] found that the hydrophilic of nature of oil palm EFB is the main reason for the higher amount of water absorption at any fiber loading in oil palm EFB composites. In addition, due to the higher proportion of hemicellulose, oil palm EFB fiber presents a higher water absorption and hydrophilic nature [18]. Hence, the addition of kenaf fiber into oil palm EFB fiber lead to decreased water absorption caused by the packed arrangement of hybrid composites due to the lower hydrophilic nature of kenaf fiber compared to oil palm EFB fiber.

### 4.7. Thickness Swelling

**Figure 6:**
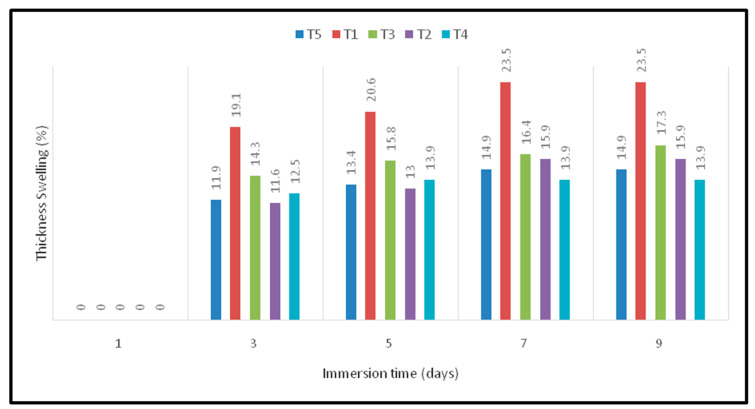
Thickness swelling (%) of oil palm EFB/kenaf woven fiber-reinforced epoxy based bi-layer composites.

The dimensional stability of composites can be measured through thickness swelling experiment. When a fiber-reinforced polymeric composite is exposed to different temperatures and humidity levels, the induced stress affects the fiber/matrix interface and leads to the decomposition of the composite samples. On the other hand, prolonged water uptake increases the thickness and affects the dimensions of composites [32]. A thickness swelling experiment was performed for several days until the sample reached constant weight. Figure 6 indicates the thickness swelling of oil palm EFB/woven kenaf fiber-reinforced epoxy composites. From the observation, oil palm EFB (T1) composites show the highest value among this type of composite. Moreover, it has been found that an increase in water immersion time increases the thickness swelling values. With an increase in the exposure time of the composite sample, fiber swelling was observed. This is mainly due to the hydrophilic nature of the natural fiber. From Figure 6, it is clear that T5 (oil palm FFB composites) samples showed a higher water uptake and thickness swelling. In contrast, T1 (kenaf composites) samples exhibited moderate water absorption and thickness swelling. The hybridization of oil palm EFB and kenaf fibers drastically reduces the water uptake and thickness swelling. Furthermore, it is attributed to the exposure of lignocellulosic fiber on the surface of the hybrid composite [18].

## 5. Conclusions

In this study, the influence of hybridizing oil palm EFB mats and woven kenaf fabric on mechanical, physical and morphological properties was studied. The following conclusions were drawn from the findings:

- The tensile and flexural properties of the oil palm EFB/kenaf woven fiber-reinforced epoxy-based bi-layer hybrid clearly indicate that the EFB fiber failed to act as a major reinforcement for the hybrid composites, while T4 hybrid composites (EFB (15 wt%):Kenaf (35 wt%) showed an excellent performance with better tensile and flexural properties.
- Poor interfacial bonding between EFB fiber and the epoxy matrix (T1) is the main factor that reduces the stress transfer and load-carrying capability, subsequently lowering the strength of the material. SEM evidence showed several poor fiber distributions and fiber pullouts in the composites.
- Nonetheless, among the hybrid composites, the T2 composites ((EFB (35 wt %):Kenaf (15 wt %)) exhibited a better impact resistance.
- Due to their random orientation, oil palm EFB-based composites (EFB (50 wt %):Kenaf (0 wt %)) showed a higher void content, water absorption and thickness swelling behavior.
- Overall, these hybrid composites can be used in non-load bearing applications. Moreover, the epoxy resin can be replaced with a biodegradable polymer to produce green and sustainable composites without sacrificing the performance.

## Figures and Tables

**Table 1:** Oil palm fiber and kenaf properties [21,22].

Properties	Oil Palm Fiber	Kenaf Fiber
Density (g/cm^3^)	1.15	1.26
Tensile strength (MPa)	71	282.60
Tensile modulus (GPa)	1.7	7.13
Elongation at break (%)	11	5–9
Cellulose content (%)	49.6	66.89
Lignin content (%)	21.2	6.85

**Table 2:** Formulations of oil palm empty fruit bunch (EFB)/kenaf woven fiber-reinforced epoxy-based bi-layer hybrid composites.

Type of Composites	Epoxy Resin (wt%)	EFB (wt%)	Kenaf (wt%)
Pure EFB(T1)	50	50	0
EFB/kenaf(T2)	50	35	15
EFB/kenaf(T3)	50	25	25
EFB/kenaf(T4)	50	15	35
Pure kenaf (T5)	50	0	50

**Table 3:** ANOVA test for tensile modulus of five samples.

Source	SS	Df	MS	F-ratio	*p*-Value
BG	754,782.87	3	251,594.29	1.8446	0.0000
WG	2,180,535.34	16	136,283.46		

Abbreviations: between groups (BG), within group (WG), sum of square (SS), degree of freedom (Df), mean square (MS).

**Table 4:** ANOVA test for tensile strength of five samples.

Source	SS	Df	MS	F-ratio	*p*-Value
BG	884.35	3	294.78	17.91	0.0000
WG	263.42	16	16.464		

**Table 5:** ANOVA test for flexural modulus of five samples.

Source	SS	Df	MS	F-ratio	*p*-Value
BG	62,151,967.86	3	20,717,322.62	297.30	0.000
WG	1,114,984.69	16	69,686.54		

Analysis of variance (ANOVA) test for tensile modulus of five samples.

**Table 6:** ANOVA test for flexural strength of 5 samples.

Source	SS	Df	MS	F-ratio	*p*-Value
BG	22,384.07	3	7461.36	77.02	0.0000
WG	1550.08	16	96.88		

**Table 7:** ANOVA test for impact strength of five samples.

Source	SS	Df	MS	F-ratio	*p*-Value
BG	12.38	3	4.1256	5.19	0.011
WG	12.73	16	0.7955		

**Table 8:** Void content and density of oil palm EFB/kenaf woven fiber-reinforced epoxy-based hybrid composites.

Composites	Void Content (%)	Density (g/cm^3^)
Pure EFB (T1)	8.40	1.02
EFB: Kenaf (1:4) (T4)	4.20	1.15
EFB: Kenaf (1:1) ((T3)	6.40	1.08
EFB:Kenaf (4:1) ((T2)	3.00	1.10
Pure Kenaf (T5)	2.21	1.40
